# Atypical hemolytic uremic syndrome triggered by mRNA vaccination against SARS-CoV-2: Case report

**DOI:** 10.3389/fimmu.2022.1001366

**Published:** 2022-09-28

**Authors:** Romana Rysava, Martina Peiskerova, Vladimir Tesar, Jan Benes, Martin Kment, Ágnes Szilágyi, Dorottya Csuka, Zoltán Prohászka

**Affiliations:** ^1^ Department of Nephrology, First Faculty of Medicine, General University Hospital, Charles University, Prague, Czechia; ^2^ University Hospital, Charles University – Faculty of Medicine, Hradec Králové, Czechia; ^3^ Department of Anesthesiology, Perioperative Medicine and Intensive Care, Masaryk Hospital, Jana Evangelisty (JE) Purkinje University, Ústi nad Labem, Czechia; ^4^ Department of Clinical and Transplant Pathology, Institute of Clinical and Experimental Medicine (IKEM), Prague, Czechia; ^5^ Department of Internal Medicine and Hematology, Semmelweis University, Budapest, Hungary; ^6^ Research Group for Immunology and Haematology, Eotvos Lorand Research Network (Office for Supported Research Groups), Semmelweis University, Budapest, Hungary

**Keywords:** atypical hemolytic uremic syndrome, thrombotic microangiopathies, acute kidney injury, mRNA vaccine against SARS-CoV-2, complement, genetic risk, eculizumab, ravulizumab

## Abstract

Atypical hemolytic uremic syndrome (aHUS), also called complement-mediated hemolytic uremic syndrome (CM-HUS), is a rare disease caused by dysregulation in the alternative complement activation pathway. It is a life-threatening condition causing ischemia of a number of organs, and it typically causes acute kidney injury. This disorder may be triggered by various factors including viral or bacterial infections, pregnancy, surgery, and injuries. In about 60% of cases, the genetic origin of the disease can be identified—commonly mutations affecting complementary factor H and MCP protein. Eculizumab, a monoclonal antibody to the C5 component of the complement, represents the current effective treatment.We describe a case of a young woman with a previous history of polyvalent allergies, who developed atypical hemolytic uremic syndrome after vaccination with mRNA vaccine against SARS-CoV-2. The disease manifested by scleral bleeding, acute renal insufficiency, anemia, and thrombocytopenia. The patient was treated with *plasma exchanges* without sufficient effect; remission occurred only after starting treatment with eculizumab. Genetic examination showed that the patient is a carrier of multiple inherited risk factors (a rare pathogenic variant in *CFH*, MCPggaac haplotype of the *CD46* gene, and the risk haplotype *CFH* H3). The patient is currently in hematological remission with persistent mild renal insufficiency, continuing treatment with eculizumab/ravulizumab. By this case report, we meant to point out the need for careful monitoring of people after vaccination, as it may trigger immune-mediated diseases, especially in those with predisposing factors.

## Introduction

Thrombotic microangiopathies (TMAs) are conditions that manifest themselves in mechanical hemolytic anemia (negative Coombs test) with the presence of reticulocytosis and schistocytes in the blood smear and thrombocytopenia (1). They include several groups of diseases, the most important and common of which are thrombotic thrombocytopenic purpura (TTP); TMA associated with pregnancy [HELLP syndrome (Hemolysis, Elevated Liver enzymes and Low Platelets), preeclampsia, or acute fatty liver of pregnancy (AFLP)]; hemolytic-uremic syndrome (HUS) associated with infection, which particularly includes STEC-HUS (caused by infection of *E. coli*-producing Shiga toxin) and neuraminidase HUS (in pneumonia caused by strains of *Streptococcus pneumoniae*-producing neuraminidase); or infections such as HIV, influenza, and SARS-CoV-2. Secondary forms of HUS (sHUS) represent an extremely heterogeneous group of diseases causing TMA, dominated mainly by hemolysis and diffuse endothelial damage; renal failure is not so common here. sHUS can be induced by generalized tumors, malignant hypertension, some autoimmune diseases (scleroderma, antiphospholipid syndrome, and systemic lupus erythematosus), conditions after transplantation of solid organs or bone marrow, or administration of certain drugs (e.g., some cytostatics, immunosuppressants, and antiplatelet agents) (2).

Atypical HUS (aHUS), which is now recommended to be referred to as complement-mediated HUS (CM-HUS), is caused by dysregulation in the alternative complement activation pathway. It accounts for only about 1% of all TMAs and is one of the very rare diseases with an incidence of approximately 0.5 cases per million per year (3). aHUS is a life-threatening disease causing ischemia of a number of organs with their subsequent dysfunction, typically with acute kidney injury. This disorder may be triggered by various factors including viral or bacterial infections, pregnancy, surgery, and injuries. Genetic predisposition of the disease can be identified in about 60% of cases (3, 4) and most often mutations affect the genes encoding complement factor H (*CFH*) and membrane cofactor protein (MCP, *CD46*). Eculizumab, a monoclonal antibody to the C5 component of the complement, represents the current effective and safe treatment (5).

In this report, we describe a case of a young woman who developed atypical hemolytic uremic syndrome after vaccination with mRNA vaccine against SARS-CoV-2. The disease manifested by scleral bleeding, acute renal insufficiency, anemia, and thrombocytopenia. Genetic examination showed that the patient is a heterozygous carrier of various risk factors (a rare pathogenic variation in *CFH*, MCPggaac risk haplotype of the *CD46* gene, and the risk haplotype *CFH* H3).

## Methods

All the information for this case report was gathered from the medical notes, laboratory examinations, and the hospital’s electronic systems of the following institutions, between September 2021 and March 2022: Department of Nephrology, First Faculty of Medicine, Charles University in Prague, Department of Anesthesiology, Masaryk Hospital, J.E. Purkinje University, Usti nad Labem and Semmelweis University, and Department of Internal Medicine and Hematology, Budapest. The informed consent was obtained from the patient and her parents, and the personal data were anonymized according to the local hospital policy.

Analysis of complement parameters: Blood samples were sent to the Complement Diagnostic Laboratory of Department of Internal Medicine and Hematology, Semmelweis University for the analysis of complement parameters. Activity of the classical or the alternative complement pathway was determined *via* hemolytic test or by functional commercial ELISA test (Wieslab, SVAR, Malmö, Sweden), respectively. Complement C3 and C4 levels were detected by immunoturbidimetry (Beckman Coulter, Brea, CA). Concentrations of complement factors I and B were measured by radial immunodiffusion using specific polyclonal antibodies, whereas levels of factor H, antigenic C1q, and IgG autoantibodies against factor H and C1q were determined *via* homemade ELISAs (6, 7). Concentrations of C3a and the sC5b-9 complex were determined using commercial kits (MicroVue, Quidel, San Diego, CA, USA), according to the manufacturer’s instructions.

Molecular genetic analysis: Genomic DNA was isolated from peripheral blood samples by the previously described salting-out method (8). In order to screen the coding regions of the disease-associated genes encoding factor H (CFH), factor I (CFI), membrane cofactor protein (CD46), complement component C3 (C3), factor B (CFB), thrombomodulin (THBD), and complement factor H-related protein 5 (CFHR5), PCR amplification was followed by bidirectional DNA sequencing, as described previously (9). Furthermore, multiplex ligation-dependent probe amplification (MLPA) was performed by using the SALSA MLPA P236-B1 probemix (MRC Holland, Amsterdam, The Netherlands), in order to detect large deletions or duplications in the CFH, CFHR1, CFHR2, CFHR3, CFHR4, and CFHR5 genes.

## Case description

The 21-year-old woman was admitted to the local internal medicine department due to the development of sclera hematomas and the detection of thrombocytopenia, both of which appeared within 24 h after the administration of a second dose of mRNA vaccine against SARS-CoV-2 (Comirnaty). The patient had a history of idiopathic epilepsy temporarily treated with carbamazepine and ovary resection for teratoma. She also reported polyvalent allergies to ibuprofen, pethidine, penicillin, cefuroxime, and wasp stings. She has not been pregnant yet, and she did not take hormonal contraceptives.

Upon admission to the hospital, in addition to thrombocytopenia, the patient also experienced non-oliguric acute renal failure (AKI), grade 2 according to KDIGO (S-creatinine with a maximum of 2.42 mg/dl), hyperbilirubinemia (2.96 mg/dl), progressive anemia with a decrease in hemoglobin from 12.2 to 7.8 g/dl within 48 h and the presence of schistocytes in the smear from peripheral blood counts (42/1,000 erythrocytes), and elevation of lactate dehydrogenase. TMA was suspected and two pulses of methylprednisolone at a dose of 500 mg and two units of frozen plasma were administered. Within 2 days, the patient was taken to a higher-level hospital to start plasma exchanges, as the platelets dropped to 26 × 10^3^/µl. VITT (vaccine-induced immune thrombocytopenia; another term used VIPIT—vaccine-induced prothrombotic immune thrombocytopenia) was established as a working diagnosis; however, antibodies to the PF4–heparin complex (HIT antibody) were negative. The value of ADAMTS13 activity was within the normal range, STEC infection and Shiga toxin production were not demonstrated, and expression of MCP on granulocytes was within the normal range. The patient underwent a total of 15 plasma exchanges, during which platelets improved (up to 92 × 10^3^/µl) and TMA symptoms declined, but renal function did not improve. After plasma exchange discontinuation, platelets decreased again, together with anemia and nephrotic proteinuria (10 g/day) development. On the 18th day since the beginning of the health disturbances, and after consulting with our tertiary care center, eculizumab therapy was initiated, and later, on the 21st day after the first symptoms of the disease appearance, the patient was transferred to our nephrology department.

**Figure 1:**
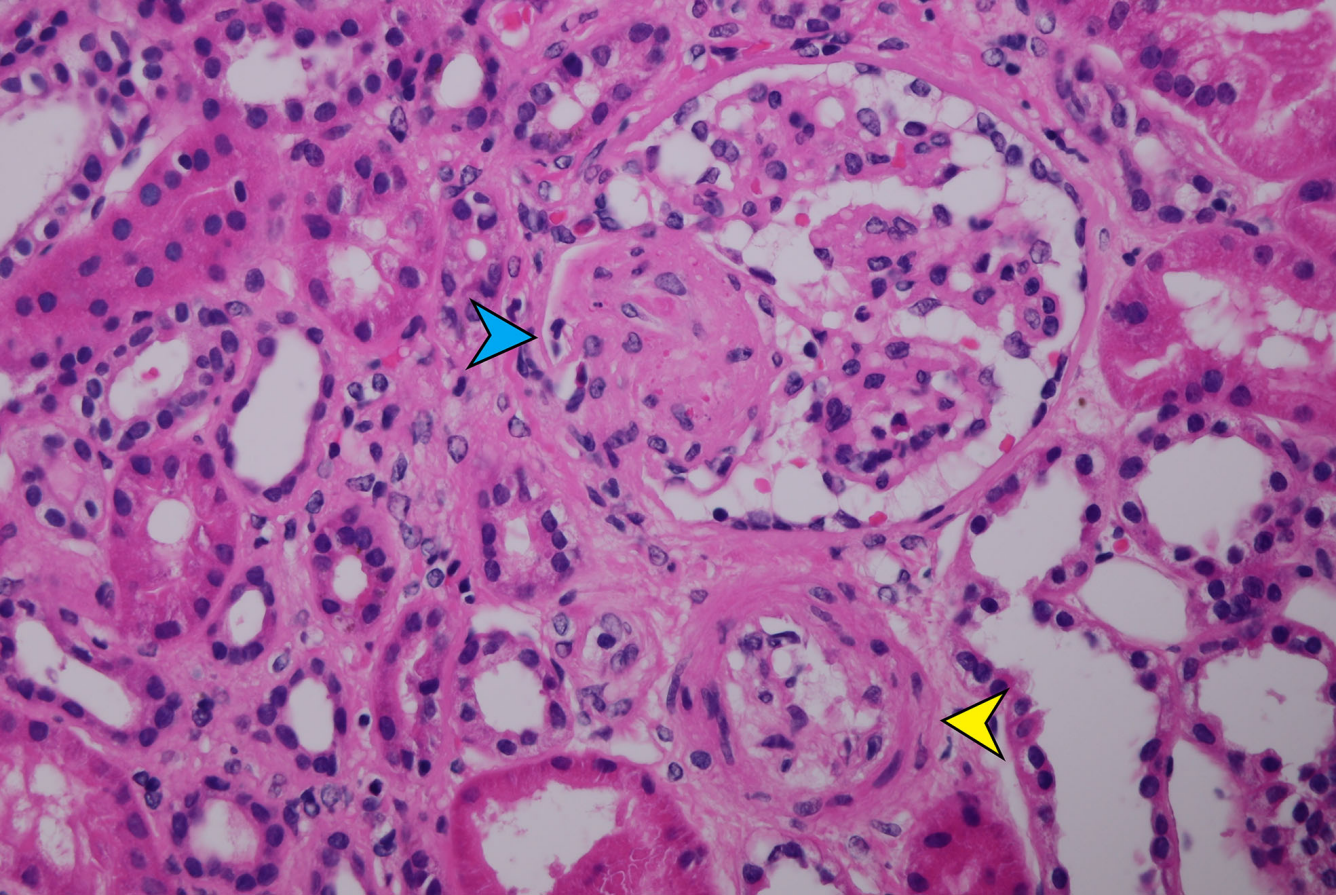
Segmental microthrombosis and mesangiolysis with entrapped erythrocytes (blue arrow) and arteriole with narrowed lumen due to marked intimal edema (yellow arrow) (HE-400×).

**Figure 2:**
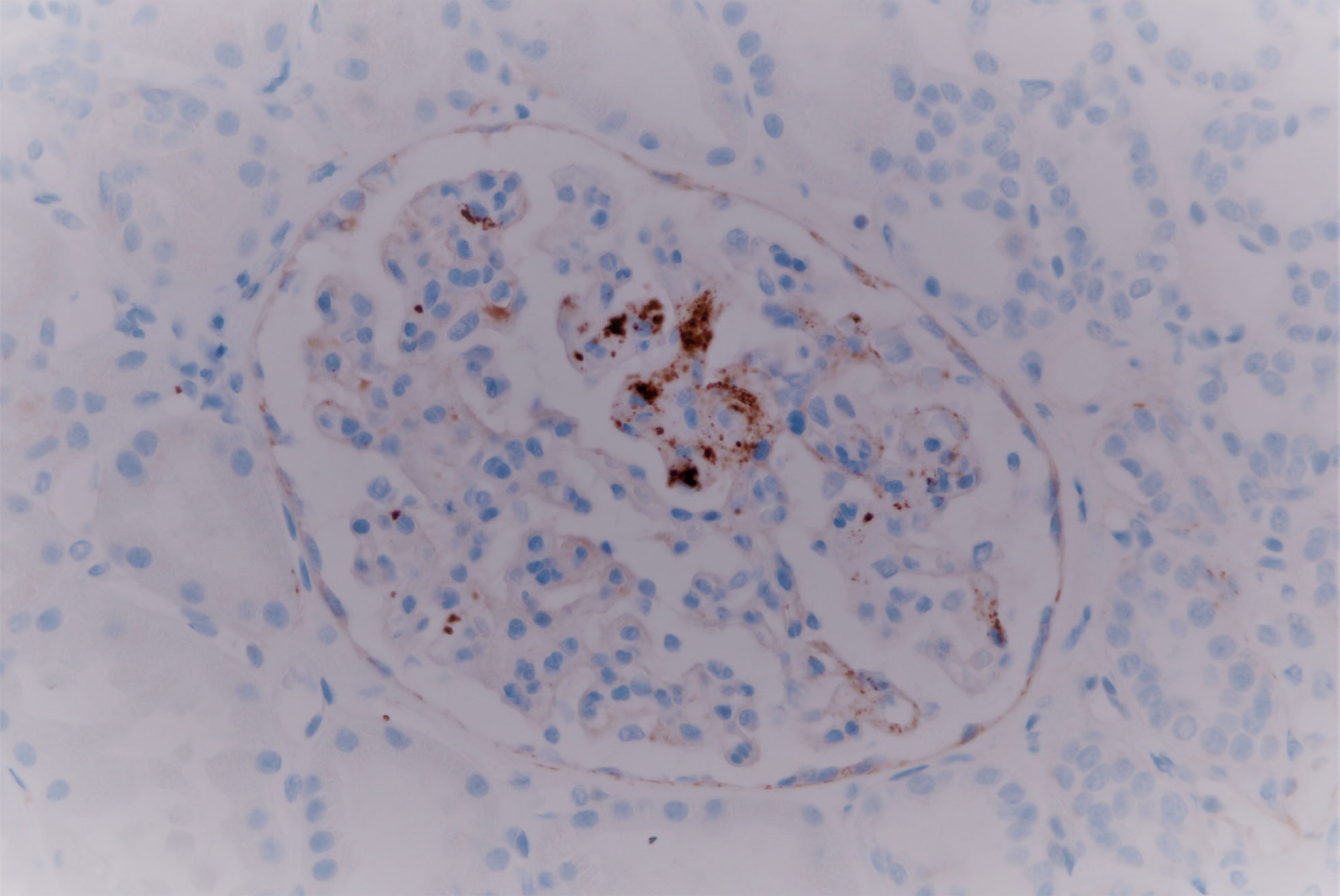
Segmental microthrombosis is highlighted by immunostain for CD61 (platelet-specific glycoprotein) (CD61-400×).

At admission, the patient was significantly hyperhydrated with swelling of the lower extremities up to the groin. Severe arterial hypertension was borderline corrected by a five-drug combination, in which, however, RAAS blockers and diuretics were missing. Laboratory parameters at admission are presented in the **Table 1**. All autoantibodies, including anti-Factor H, were negative, while the level of C3 complement component was reduced to even 55 mg/dl (normal range, 90–180). With ongoing eculizumab treatment, after stabilization of blood pressure and platelet normalization, a renal biopsy was performed (1 month after the onset of symptoms), which showed signs of prolonged TMA with thrombi in 7 of 16 examined glomeruli, interstitial edema with incipient fibrosis, and dilation of part of the tubules due to intraluminal accumulation of uromodulin. None of the 16 glomeruli found have been obsolescent yet (**Figures 1** and **2**). Subsequent comprehensive genetic examination involving the analysis of the genes encoding complement factor H (*CFH*), factor I (*CFI*), factor B (*CFB*), C3 (*C3*), membrane cofactor protein (*CD46*), thrombomodulin (*THBD*), and complement Factor H-Related Protein 5 (*CFHR5*) showed that the patient is a heterozygous carrier of a rare pathogenic variation in *CFH* (c.3096C>A, p.C1032X) and also heterozygous for the MCPggaac risk haplotype of the *CD46* gene, which is reported as a risk factor of developing aHUS (10, 11). At the same time, the heterozygous carrier state of the risk haplotype *CFH* H3 was deduced based on the presence of the following *CFH* polymorphisms: heterozygous for the c.-331C>T (rs3753394) polymorphism, and homozygous for the rare alleles of the Q672Q (rs3753396) and E936D (rs1065489) polymorphisms. The patient also carried a rare variation in heterozygous form in the *CFHR5* gene (c.432A>T, p.K144N) that was considered as likely benign. Altogether, these findings support the diagnosis of complement-mediated aHUS.

**Table 1:** Timeline of the laboratory results in our patient with CM-HUS before and after the eculizumab therapy initiation.

Parameter (units)[normal range]	1.9.2021 before starting eculizumab	1.10.2021 after 2 doses of eculizumab	1.1.2022 on eculizumab	1.3.2022 on eculizumab
Hemoglobin (g/dl) [12.0–16.0]	7.9	9.5	13.7	13.8
Platelets (10^3^/µl) [150.0–400.0]	71	147	186	183
Lactate dehydrogenase (U/L) [100.0–190.0]	360	410	320	230
Haptoglobin (mg/dl) [35–200]	<6	<6	186	180
Schistocytes (per mille) [0.0–10.0]	10	33	0	1
Serum creatinine (mg/dl) [0.5–1.18]	2.84	2.59	1.64	1.5
eGFR (CKD–EPI) (ml/min/1.73 m^2^) [90.0–120.0]	22.8	25.8	44.4	49.2
Proteinuria (PCR) (mg/g) [0.0–150.0]	740	160	80	90
Complement C3 (mg/dl) [90–180]	65	55	72	90
ADAMTS13 metalloprotease activity (%) [67–150]	73	49	NA	NA
Total complement activity, classical pathway (CH50/ml) [48–103]	61	8	NA	NA
Total complement activity, alternative pathway (%) [70–125]	114	0	NA	NA
Complement C4 (mg/dl) [15.0–55.0]	38	25	NA	NA
Factor H antigen (mg/dl) [25.0–88.0]	18.7	14.6	NA	NA
Factor I antigen (%) [70–130]	100	61	NA	NA
Factor B antigen (%) [70–130]	121	47	NA	NA
C1q antigen (mg/dl) [6.0–18.0]	11.8	8.3	NA	NA
sC5b-9 (terminal complement complex) (ng/ml) [110–252]	NA	219	NA	NA
Anti-C1q IgG autoantibody (U/ml) [<52]	2	NA	NA	NA
Anti-factor H IgG autoantibody (AU/ml) [<110]	16	NA	NA	NA
C3-nephritic factor (%) [<10%]	6	5.4	NA	NA
Serum-free eculizumab (µg/ml)	0	159	221	NA

NA, Not available.

Due to the severity of the disease, which developed after vaccination, the patient’s family was screened for the identified variations. The mother (born in 1974) and two younger brothers (born in 2003 and 2011) of the patient also carried the rare pathogenic variation, *CFH* p.C1032X. The mother does not bear any of the other studied variations, while the brothers carried risk variations similar to our patient (heterozygous MCPggaac and heterozygous *CFH* H3 or E936D). The mother and both brothers are healthy. The mother and the older brother born in 2003 have been vaccinated with two doses of the same vaccine [mRNA vaccine against SARS-CoV-2 (Comirnaty)]; the younger brother born in 2011 has never been vaccinated against SARS-CoV-2.

**Figure 3:**
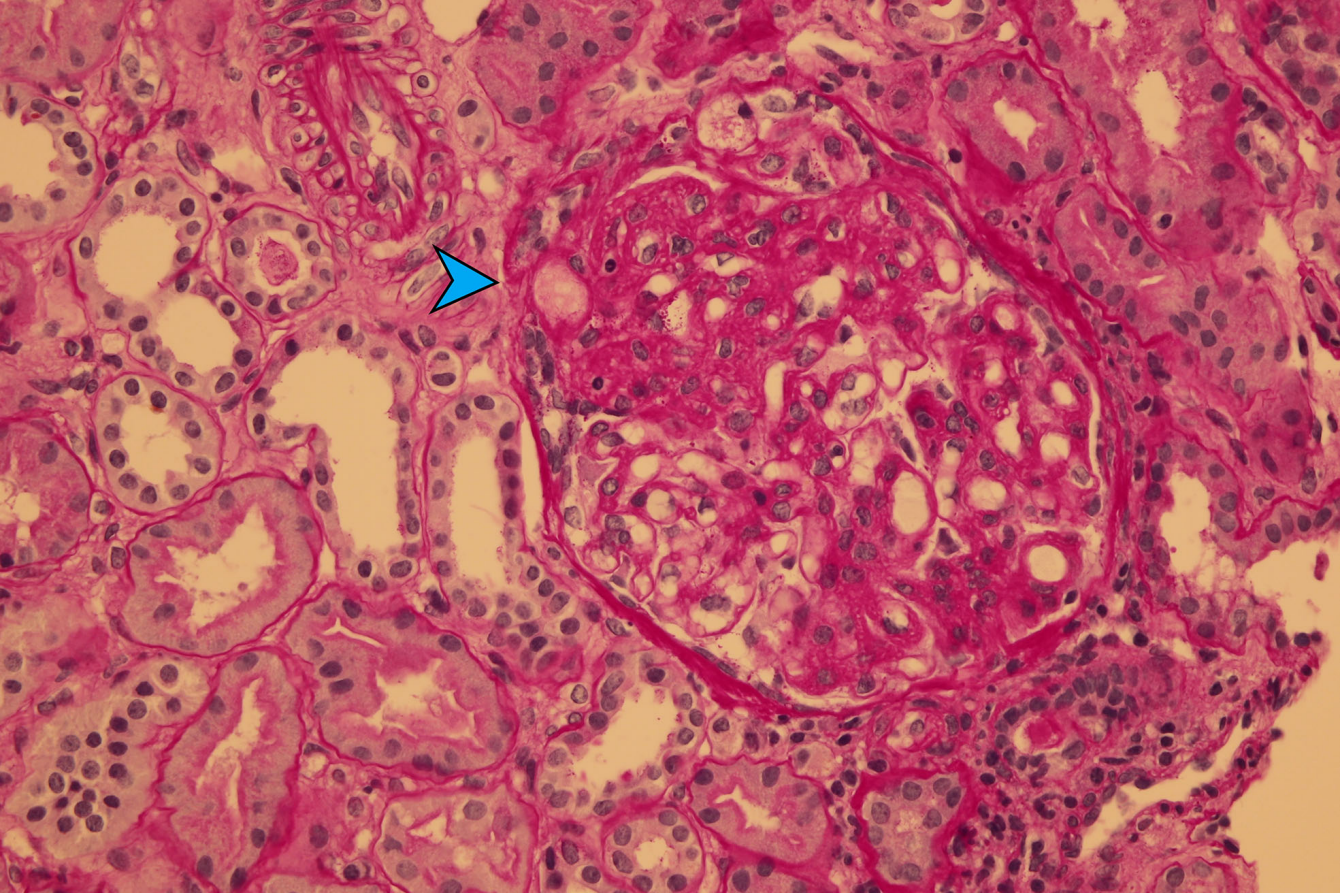
Chronic TMA represented by segmental glomerulosclerosis (blue arrow) (PAS-400×).

In view of all the above findings, the patient was continued to be treated with eculizumab (14 doses, from October 2021 to March 2022) and then switched to ravalizumab (a similar drug with a longer half-life, administered every 8 weeks, from March 2022 up to now) with a good effect and overall improvement in the condition. Nevertheless, TMA symptoms completely disappeared only after a few weeks of treatment, and mild renal dysfunction persisted. Therefore, a second renal biopsy was performed 4 months later (February 2022) with a similar finding as in the first, but with a progression of tubulointerstitial fibrosis to 35% and signs of already present tubular atrophy (**Figure 3**). Vaccination against meningococcus (Menveo and Bexsero), which is required for treatment with eculizumab, was carried out without major complications.

## Discussion

According to the published data from 2006 to 2013, during the first attack of aHUS disease, the risk of developing end-stage renal disease (ESRD) or death was 33%–40% (12, 13). The risk of permanent kidney damage, kidney failure, or death within the first year of diagnosis was approximately 65% without treatment (12, 13). This unflattering prognosis is often not affected by treatment with plasma exchanges (PE) or fresh frozen plasma infusions, both of which are among the first treatment measures. Even in the case of our patient, this treatment had only a partial and transient effect, and after the termination of PE, the signs of TMA returned again. A major breakthrough in the treatment of aHUS was a monoclonal antibody directed against the C5 component of the complement, eculizumab, which prevents the formation of C5a and C5b-9 components of the complement. This therapy has shown a positive effect on stopping complement activation and stabilizing or improving renal function in patients with aHUS (14, 15). The novel product, ravulizumab, shows a comparable therapeutic effect with eculizumab in patients with aHUS, with the advantage of longer half-life and thus the possibility of administering infusions with a longer time interval.

In the case presented here, treatment of the patient with eculizumab was started relatively late, only on the 18th day after the onset of the first symptoms of the disease, which may certainly contribute to the delayed response to treatment and incomplete repair of renal function. From the work of Van de Walle et al., we know that better renal survival is found in individuals whose treatment with complement blockers is started within 7 days of the first symptoms of the disease (16). Unfortunately, in our patient, a number of active changes described in the first renal biopsy have passed into chronic changes, which was confirmed by the second renal biopsy. Another reason we considered in the slow response to treatment may have been a reduced level of eculizumab due to a massive proteinuria and possible leakage of the drug into the urine. This could lead to an insufficient blockade of alternative pathway of complement activity. Nevertheless, the measured serum-free eculizumab levels, as well as examinations focused on classical and alternative pathways of complement activity, showed therapeutic serum levels of the drug (159 and 221 μg/ml, respectively) and sufficient blockade of both pathways. Based on that, this possible reason was therefore excluded.

COVID-19 infection is very often associated with the development of AKI, especially in individuals requiring a stay in intensive care units (17). There may be several reasons for the development of AKI, but dehydration in diarrhea or hypoperfusion of the kidneys in cardiorespiratory failure is predominantly applied. Often, the overuse of NSAIDs to control temperature and manifestations of viral diseases may also contribute to AKI. Like other viral diseases, COVID-19 can act as a trigger for *de novo* induced glomerular diseases. However, there are only a few cases where a COVID-19 infection would cause aHUS or its relapse (18, 19).

Not only the COVID-19 infection itself, but also vaccination against SARS-CoV-2 may contribute to various renal diseases, as many of such cases have been reported in the literature. Several cases of *de novo* ANCA-associated vasculitis have been reported after various types of vaccines (mRNA—Moderna or AZD1222—Oxford-AstraZeneca) (20–22), as well as *de novo* membranous glomerulonephritis (23) or its relapse (24). Relapse of the disease has also been reported in a patient with lupus nephritis (25). Other diseases that can develop after vaccination against SARS-CoV-2 include lgA nephropathy (26) or scleroderma renal crisis (27).

The development of aHUS after the use of a vector COVID-19 vaccine (ChAdOx1 nCoV-19, AZD1222) has been reported in the literature in only one case so far (28). Of course, for all these case reports, it is not possible to decide unequivocally whether it is a causal connection or just a random confluence of the two things. However, activation of the immune system caused by any type of vaccination can very likely play a role as a trigger for immune-mediated diseases, especially in those with predisposing factors.

In differential diagnosis, VITT was considered in our patient (29). This rare complication occurs after vaccination with adenovirus vaccines with a frequency of 3–10/million vaccinated (30). It usually arises after the first dose of the vaccine and can manifest itself in thrombocytopenia, thrombosis in the cerebral venous sinus or in splanchnic veins, thromboembolism into the cerebral arteries, or signs of TMA. The vast majority of patients have thrombocytopenia, D-dimer elevation, and positive antibodies to platelet factor 4 (anti-PF4 antibody). This diagnosis was subsequently ruled out in our patient for a number of reasons: the patient was vaccinated with an mRNA vaccine, had no thrombotic events, and anti-PF4 antibodies were negative. Despite complex treatment with anticoagulation, administration of high-dose immunoglobulins, or eculizumab, not all patients may experience complete recovery (31).

The described case report points to the fact that not only COVID-19, but also vaccination against it can act as a trigger for the development of aHUS especially in those subjects who carry underlying aHUS-associated mutations and/or risk polymorphisms. Differential diagnosis is not always easy and must include the syndromes described above, such as VITT. This case report is by no means intended to call into question the enormous benefits of vaccination against the SARS-CoV-2 virus, which has already prevented the deaths or severe complications of COVID-19 infection in millions of people. It is only intended to point out the need of a careful monitoring of the individuals after vaccination, as especially in those with pre-existing predisposing factors, rare immune-mediated disorders may be triggered. We think that our report may draw attention to healthcare providers or even the general population to quickly identify possible manifestations and adverse outcomes that could be related to COVID-19 vaccination.

We are aware that even though the major symptoms, signs, and laboratory changes occured within 24 h of administration of the vaccine (which supports the idea of casuality), we cannot exclude the possibility of two random events, which we consider to be the main limitation of our case report. However, we think that we appropriately excluded the other possible causes of adverse events related to vaccine, especially VITT with anti-PF4 analysis, renal biopsy, and also genetic study of the patient and her family. We present the review of published data that can help readers to better understand the case.

## Data availability statement

The raw data supporting the conclusions of this article will be made available by the authors, without undue reservation.

## Author contributions

RR is the professor of Nephrology and the main author and corresponding author of the manuscript. ZP is the professor of Immunology, responsible for complement and genetic laboratory results and is a corresponding author too. He critically revised the article for important intellectual content. VT, MP, JB, MK, AS, and DC are physicians and researchers, substantial contributors to the conception or design of the work, or the acquisition, analysis, or interpretation of data for the work. All authors contributed to the article and approved the submitted version.

## Funding

This study was supported by the research project of Charles University: COOPERATIO Internal Disciplines and the research grant of the General Teaching Hospital RVO-VFN 64165. This study was supported by the Higher Education Institutional Excellence Program of the Ministry of Human Capacities in Hungary, within the framework of the molecular biology thematic program of the Semmelweis University, by the TKP2021-EGA-24 (MOLORKIV) and by the 2020-1.1.6-JOVO421 2021-00013 grants to ZP.

## Conflict of interest

The authors declare that the research was conducted in the absence of any commercial or financial relationships that could be construed as a potential conflict of interest.

## Publisher’s note

All claims expressed in this article are solely those of the authors and do not necessarily represent those of their affiliated organizations, or those of the publisher, the editors and the reviewers. Any product that may be evaluated in this article, or claim that may be made by its manufacturer, is not guaranteed or endorsed by the publisher.

## Abbreviations

ADAMTS13, A** ***d*** *isintegrin* ***a*** *nd* ***m*** *etalloproteinase with a* ***t*** *hrombo* ***s*** *pondin type 1 motif, member* ***13***—also known as *von Willebrand factor-cleaving protease;* aHUS, Atypical hemolytic uremic syndrome (synonym of CM-HUS);ANCA vasculitis, Anti-neutrophil cytoplasmic antibody; Anti-PF4 antibodies, Antibodies to platelet factor 4; AFLP, Acute fatty liver of pregnancy (preeclampsia); AKI, Acute kidney injury; CFH, Complement factor H; CM-HUS, Complement-mediated hemolytic uremic syndrome (synonym of aHUS); HELLP syndrome, Hemolysis, Elevated Liver enzymes and Low Platelets; HIT antibodies, Heparin-induced thrombocytopenia; KDIGO guidelines, Kidney Disease: Improving Global Outcomes; MCP protein, Membrane cofactor protein*;* NSAIDs, Non-steroidal anti-inflammatory drugs, PF4, https://www.nature.com/articles/s41420-019-0188-0; TMA, Thrombotic microangiopathy; TTP, Thrombotic thrombocytopenic purpura; STEC-HUS, Hemolytic–uremic syndrome associated with infection, in particular caused by *E. coli*-producing Shiga toxin; VIPIT, Vaccine-induced prothrombotic immune thrombocytopenia; VITT, Vaccine-induced immune thrombocytopenia

## Glossary

**Table:**

ADAMTS13	A* * ***d*** *isintegrin * ***a*** *nd * ***m*** *etalloproteinase with a * ***t*** *hrombo* ***s*** *pondin type 1 motif, member * ***13***—also known as *von Willebrand factor-cleaving protease*
aHUS	Atypical hemolytic uremic syndrome (synonym of CM-HUS)
ANCA vasculitis	Anti-neutrophil cytoplasmic antibody
Anti-PF4 antibodies	Antibodies to platelet factor 4
AFLP	Acute fatty liver of pregnancy (preeclampsia)
AKI	Acute kidney injury
CFH	Complement factor H
CM-HUS	Complement-mediated hemolytic uremic syndrome (synonym of aHUS)
HELLP syndrome	Hemolysis, Elevated Liver enzymes and Low Platelets
HIT antibodies	Heparin-induced thrombocytopenia
KDIGO guidelines	Kidney Disease: Improving Global Outcomes
MCP protein	Membrane cofactor *protein*
NSAIDs	Non-steroidal anti-inflammatory drugs
PF4	Platelet factor 4
TMA	Thrombotic microangiopathy
TTP	Thrombotic thrombocytopenic purpura
STEC-HUS	Hemolytic–uremic syndrome associated with infection in particular caused by *E. coli*-producing Shiga toxin
VIPIT	Vaccine-induced prothrombotic immune thrombocytopenia
VITT	Vaccine-induced immune thrombocytopenia
